# Evaluating the safety and efficacy of zuranolone in the management of major depressive disorder and postpartum depression, with or without concurrent insomnia: a rigorous systematic review and meta-analysis

**DOI:** 10.3389/fpsyt.2024.1425295

**Published:** 2024-07-05

**Authors:** Adarsh Raja, Saboor Ahmed, Muhammad Basit Ali Siddiqui, Syeda Lamiya Mir, Rakesh Kumar, Muhammad Ahmed, Sandesh Raja, Shafin Bin Amin, Hafsah Alim Ur Rahman, Fnu Deepak, Muhammad Sohaib Asghar

**Affiliations:** ^1^ Department of Internal Medicine, Shaheed Mohtarma Benazir Bhutto Medical College Lyari, Karachi, Pakistan; ^2^ Department of Internal Medicine, Dow University of Health Sciences, Karachi, Pakistan; ^3^ Department of Internal Medicine, Division of Nephrology and Hypertension, Mayo Clinic, Rochester, MN, United States

**Keywords:** zuranolone, major depressive disorder, postpartum depression, insomnia, meta-analysis

## Abstract

**Introduction:**

Major depressive disorder (MDD), postpartum depression (PPD), and insomnia are neuropsychological conditions in which zuranolone is used to improve symptoms and prognosis of the disorder. This meta-analysis aimed to determine the efficacy of zuranolone in comparison to other drugs used for treating these conditions.

**Methods:**

This meta-analysis included patients aged between 18 and 75 years who were diagnosed with major depressive disorder and postpartum depression with or without insomnia and were administered zuranolone for treatment. Only randomized controlled trials (RCTs) were included, and animal studies were excluded. The databases used were PubMed, Scopus, Cochrane, and Clinicaltrials.gov, with MeSH terms and relevant keywords for (Zuranolone) and (Depression). The Cochrane risk of bias tool was used for quality assessment.

**Results:**

The meta-analysis included eight RCTs that analyzed data from 2031 patients. The meta-analysis revealed statistically significant changes in the Hamilton Depression Rating Scale (HAM-D), Montgomery-Åsberg Depression Rating Scale (MADRS), Hamilton Anxiety Rating Scale (HAM-A), and treatment-emergent adverse effects (TEAE) scores in the PPD subgroup. HAM-D and TEAEs scores were also significant in the MDD subgroup, but the changes in the MADRS, HAM-A, and Bech-6 scores were insignificant. Serious adverse events were insignificant in all subgroups.

**Conclusion:**

Meta-analysis found a significant improvement in depressive symptoms with zuranolone treatment, especially on day 15. This suggests that zuranolone is a promising therapeutic option for patients with MDD and PPD with or without insomnia.

**Systematic review registration:**

https://www.crd.york.ac.uk/prospero/display_record.php?RecordID=459554, identifier CRD42023459554.

## Introduction

Major Depressive Disorder (MDD) is a psychiatric condition characterized by persistent feelings of sadness and hopelessness, often accompanied by other symptoms such as changes in appetite, sleep disturbances, loss of interest in activities, and cognitive impairments. According to the World Health Organization, MDD is one of the most prevalent mental disorders worldwide, affecting over 300 million people of all ages (1). In the United States, it is estimated that 8.9 million adults had MDD in 2021, making it a leading cause of disability (1–3). The exact cause of MDD is not fully understood, but it is believed to be a result of complex interactions between various risk factors (4). Another mood disorder that can present with depressed mood is postpartum depression (PPD), which affects approximately 15% of new mothers after childbirth (5, 6). Factors that increase the risk of PPD include a history of depression, exposure to violence, obesity, and poor sleep quality after childbirth (7). Both MDD and PPD can also manifest as insomnia, defined as difficulty falling asleep or maintaining sleep (8). It is estimated that 10% of the global adult population suffers from chronic insomnia, while an additional 20% experience occasional symptoms (9, 10). Insomnia is closely associated with depression, and it is believed to exacerbate symptoms of depression and increase the risk of self-harm or suicide (11). Depression can also lead to insomnia by reducing the latency of REM sleep, which disrupts the body’s natural sleep-wake cycle (12). There are several options for treating MDD, such as benzodiazepines, Selective Serotonin Reuptake Inhibitors (SSRIs), and serotonin-norepinephrine reuptake inhibitors (SNRIs). Benzodiazepines promote the release of the neurotransmitter gamma-aminobutyric acid (GABA), which inhibits nerve activity in the brain. SSRIs decrease the reuptake of serotonin, resulting in the alleviation of MDD symptoms (13–15). SNRIs inhibit the uptake of monoamines within the synaptic cleft, which ultimately leads to improvement (16). Mirtazapine is beneficial in treating MDD by blocking presynaptic alpha 2 adrenergic receptors (17).

Another class of drugs, known as neuroactive steroids, has gained significant attention in addressing these conditions. Brexanolone, in its intravenous form, and allopregnanolone are essential in treating PPD by modulating gamma-aminobutyric acid (GABAa) receptors, resulting in reduced anxiety levels and improved sleep quality (18, 19). While these drugs have demonstrated promise in treating MDD, they also have numerous adverse effects. Benzodiazepines, for instance, can cause cognitive impairment, hyperexcitability, insomnia, anxiety, and seizures upon withdrawal. In contrast, approximately 30% of patients show no response to selective serotonin reuptake inhibitors (SSRIs) (20, 21). Zuranolone, a synthetic neuroactive steroid, increases GABA release by positive allosteric modulation of GABA receptors, showing potential for treating MDD and PPD (22–24). A study by Maximos et al. revealed that zuranolone improved PPD symptoms in adults and had beneficial effects on their insomnia symptoms (23). Another study demonstrated that zuranolone effectively relieved insomnia symptoms in females with anxiety and PPD (25).

Our systematic review and meta-analysis aim to determine whether zuranolone is a more effective treatment option for patients with PPD and MDD, with or without insomnia. This information can help physicians identify alternative treatment strategies for patients with MDD and PPD who may not respond to or experience severe side effects from conventional treatments. This study can prove beneficial for patients with MDD and PPD in assessing the efficacy of zuranolone in treating these disorders in individuals with concurrent insomnia symptoms.

## Methods

### Protocol and registration

This systematic review and meta-analysis was conducted in compliance with the Preferred Reporting Items for Systematic Reviews and Meta-Analyses (PRISMA) guidelines (26). Prior to the initiation of the study, it was registered in the International Prospective Register of Systematic Reviews (PROSPERO) registry under the credentials CRD42023459554 (27).

### Search strategy

An extensive search of electronic databases, including PubMed, Scopus, Clinicaltrials.gov, PsychINFO, EMBASE, and Cochrane Central Register of Controlled Trials (CENTRAL), was performed from inception until September 2023. No restrictions were imposed on time or sample size during the literature search. The following keywords were used in the search strategy along with relevant MeSH terms and Boolean operators: “Zuranolone” OR “SAGE-217” AND “depression” OR “major depressive disorder” AND “Postpartum depression.” The detailed search strategy is presented in **Supplementary Table 1**. Furthermore, the bibliometrics of the published articles on related topics and conference proceedings were examined to ensure that no articles were overlooked.

### Study selection

All articles retrieved from the search results were entered into Rayyan.ai, an online systematic review management platform (28). After removing duplicates, two investigators (S. A. and M.B.A. S.) independently screened the remaining studies in accordance with the specified inclusion and exclusion criteria. The initial screening process involved evaluating titles and abstracts to identify potentially eligible studies, which were then followed by a full-text assessment to ensure relevance based on our predetermined criteria. Any conflicts or disagreements were resolved through a consensus process or with the assistance of a third reviewer (L. M.). Studies were selected based on the following Population, Intervention, Control, and Outcomes (PICO) criteria: randomized clinical trials with parallel groups, population aged 18–75 years diagnosed with major depressive disorder (MDD) or postpartum depression (PPD) with or without insomnia, and intervention with zuranolone for treatment compared to a control group receiving placebo. We included studies reporting outcomes such as changes from baseline in the Hamilton Depression Rating Scale (HAM-D), Montgomery–Åsberg Depression Rating Scale (MADRS), and Hamilton Anxiety Rating Scale (HAM-A). Non-randomized trials, quasi-randomized trials, animal experiments, chemistry or cell line studies, conference abstracts, comments, editorials, review articles, meta-analyses, case reports, and case series were excluded, as were publications that reported duplicate data or were written in languages other than English.

### Data extraction

Data extraction was performed by two authors: (A. R. and S.R.) In the event of disagreement, a third reviewer, M. A., was consulted to reach a consensus. Information on the title, author, year of publication, and demographics, including age, sex, and sample size in each group, was extracted meticulously. Moreover, relevant characteristics, such as the HAM-D score, dosage, length of treatment, status and degree of treatment-resistant depression, treatment strategy, adverse events, and general methodological details, including study arms and crossover trials, were also included. Data on treatment outcomes, including the post-HAM-D score, post Bech-6 score, MADRS, and post-HAM-A scores, were extracted from the included studies.

### Quality assessment

Two independent authors, R. K. and A. R., assessed the risk of bias in the included studies using the Cochrane Risk of Bias (RoB 2.0) tool for randomized controlled trials (RCTs) (29). The RoB 2.0 addressed five specific domains: (1) Bias arising from the randomization process; (2) Bias due to deviations from the intended intervention; (3) Bias due to missing outcome data; (4) Bias in the measurement of the outcome; and (5) Bias in the selection of the reported results. This tool was applied to each included study, and the studies were classified as having a high, moderate, or low risk of bias based on the RoB classification. A third review author, H. A. U. R., was consulted to resolve any disagreements concerning the ROB assessment.

### Statistical analysis

Statistical analysis was conducted using a random-effects model to pool the Mean Differences (MDs) and Risk Ratios (RRs) for continuous and dichotomous outcomes, respectively, with 95% Confidence Intervals (CIs). The pooled results were graphically represented in forest plots, and the I2 statistic was used to assess the heterogeneity across studies. An I2 statistic of > 50% was considered significant, indicating high heterogeneity, whereas a value of less than 50% indicated low heterogeneity. The I2 values were interpreted according to the guidelines provided in the Cochrane Handbook for Systematic Reviews of Interventions. Statistical significance was set at P < 0.05. Funnel plots were used to visually assess publication bias, and subgroup analyses were performed to identify sources of heterogeneity in clinical outcomes. The subgroups included patients with 1) major depressive disorder, 2) postpartum depression, and 3) Insomnia in patients with major depressive disorder or postpartum depression. Statistical analysis was conducted using Review Manager (RevMan, Version 5.4.1; The Cochrane Collaboration, Copenhagen, Denmark).

## Results

**Figure 1:**
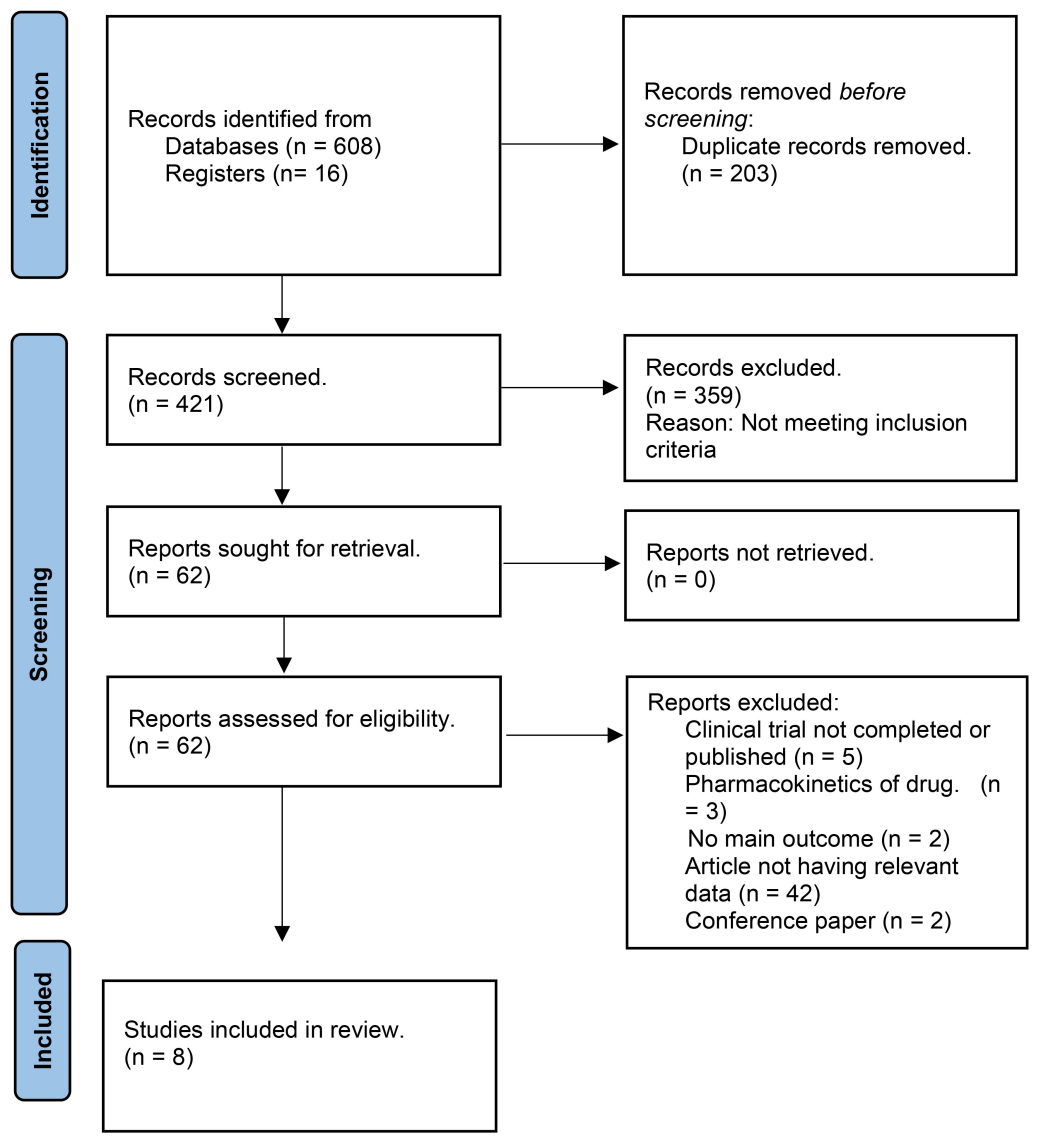
PRISMA Flowchart.

**Figure 2:**
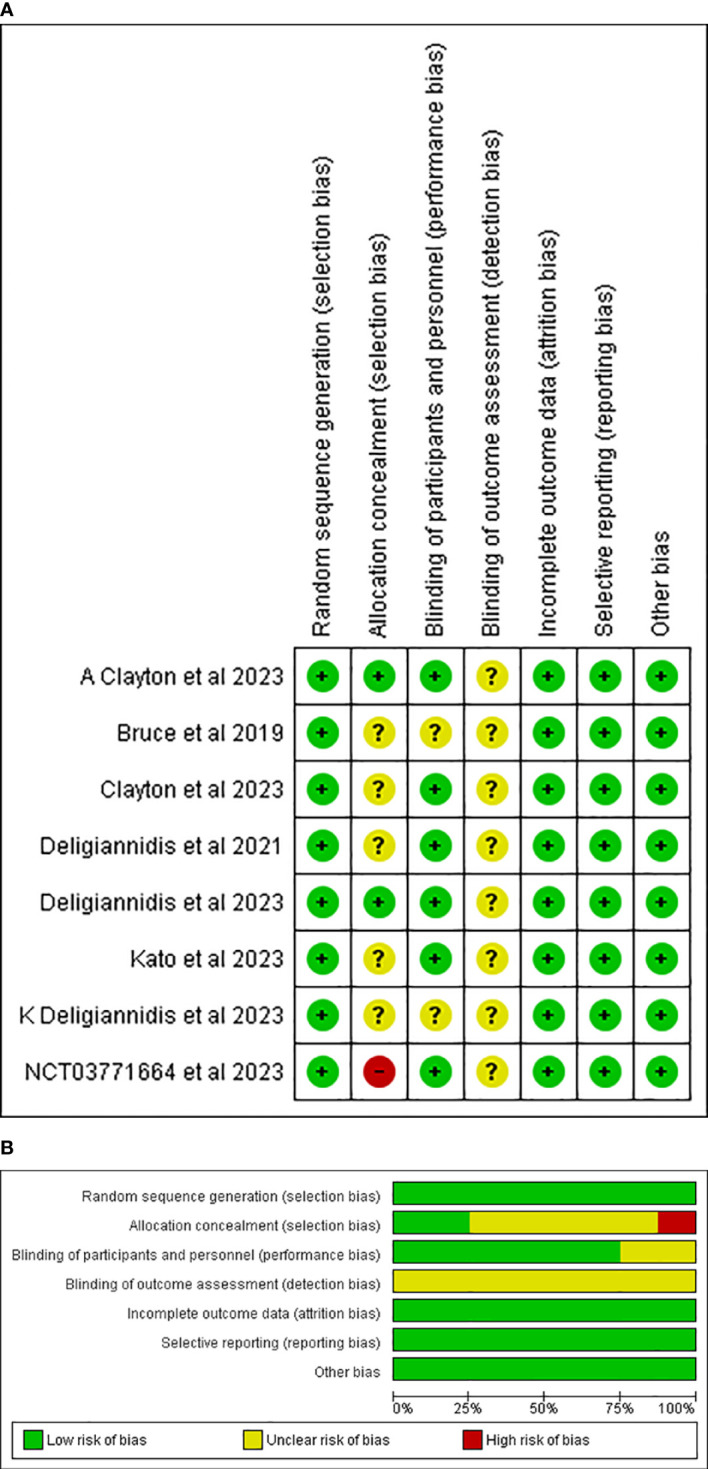
**(A)** Risk of bias summary. **(B)** Risk of bias graph.

A total of 624 studies were obtained, following which 8 RCTs were selected for inclusion in our review after excluding duplicates, reviews, and ineligible articles through Primary and Secondary screening (24, 25, 30–35). The PRISMA flowchart (**Figure 1**) provides a summary of the literature screening process. Our systematic review and meta-analysis included 8 studies that reported data on 2,031 patients. The characteristics of the included studies are presented in **Table 1**, which also indicates that all studies included had a low-to-moderate risk of bias. A detailed quality assessment of the included studies is illustrated in **Figures 2A**, **B** and detailed reasons are provided in **Supplementary Table 2**.

**Table 1:** General characteristics of included studies and patients.

Studies	Drug Dose	Patients	Total Population		Age (Years)		Gender		BMI (kg/m2)		Baseline HAM-D score (mean and sd)		Antidepressants at baseline	
							Male; Female							
			Z group	Placebo	Z group	Placebo	Z group	Placebo	Z group	Placebo	Z group	Placebo	Z group	Placebo
Gunduz-Bruce et al., 2019 (31)	zuranolone (30mg)	Major Depressive Disorder	45	44	49.1 ± 13.6	38.3 ± 12.2	20;25	14;30	30.0 ± 6.3	29.9 ± 5.2	25.2 ± 2.6	25.7 ± 2.4	12	10
Kato et al., 2023 (33)	zuranolone (20-mg)	Major Depressive Disorder	85	83	39.3 ± 12.6	40.8 ± 10.6	36;49	35;47	23.9 (4.4)	23.6 (5.3)	24.8 ± 2.4	24.5 (2.1)	NA	NA
	zuranolone (30-mg)		82		38.8 ± 12.0		37;45		22.7 (4.0)		24.6 ± 2.2		NA	
Clayton et al., 2023 (24)	zuranolone (20-mg)	Major Depressive Disorder	188	190	41.9 ± 12.2	41.4 ± 12.2	47;112	51;106	NA	NA	25.8 ± 2.8	25.8 ± 3.1	46	49
	zuranolone (30-mg)		192		42.3 ± 11.8		45;121		NA		25.9 ± 2.9		47	
Clayton et al., 2023 (32)	zuranolone (50-mg)	Major Depressive Disorder	268	269	39.4 ± 12.3	40.1± 12.6	82;186	103;166	29.6 ± 6.3	30.3 ± 6.2	26.8 ± 2.6	26.9 ± 2.7	183	190
NCT03771664 et al., 2022	zuranolone (30-mg)	Insomnia and Major Depressive Disorder	43	43	44.6 ± 12.50	42.0 ± 11.60	15;28	12;31	NA	NA	NA	NA	NA	NA
							Female							
Deligiannidis et al., 2021 (30)	zuranolone (30-mg)	Postpartum Depression	76	74	29.3 ± 5.4	27.4 ± 5.3	76	74	31.1 ± 6	30.3 ± 8	28.4 ± 2	28.8 ± 2	16	18
Deligiannidis et al., 2023 (34)	zuranolone (50-mg)	Postpartum Depression	98	98	30.0 ± 5.9	31.0 ± 6.0	98	98	30.9 ± 6.3	29.6 ± 6.3	28.6 ± 2.5	28.8 ± 2.3	15	15
Deligiannidis et al., 2023 (25)	zuranolone (30-mg)	Insomnia and Postpartum Depression	77	76	16 ± 45	16 ± 45	77	76	NA	NA	26	26	16	13

mg, miligram; NA, not available.

### Primary outcome

#### Change from baseline in HAM-D score

**Figure 3:**
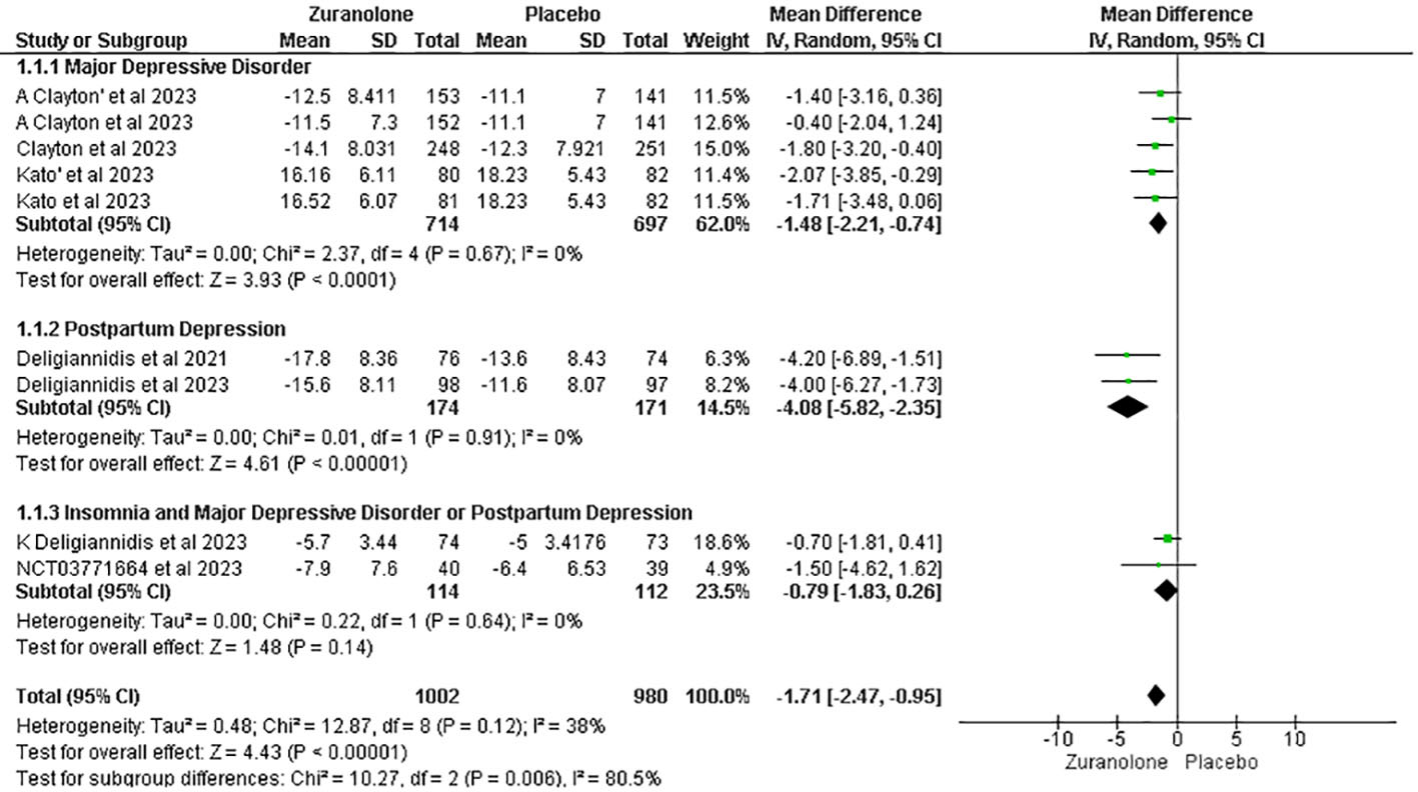
Forest plot of reduction in HAM-D score.

Seven studies were assessed to determine the relationship between changes in HAM-D scores and the use of zuranolone. The results indicated a significant decrease in the HAM-D score in zuranolone consumers compared to the placebo group (MD: -1.71; 95% CI= [-2.47, -0.95]; p<0.00001; I2 = 38%). A consistent finding was observed in the subgroup analysis for the MDD (MD: -1.48; 95% CI [-2.21, -0.74]; p<0.0001; I2 = 0%) and PPD subgroup (MD: -4.08; 95% CI [-5.82, -2.35]; p<0.00001; I2 = 0%), except for patients with insomnia and MDD or PPD (MD: -0.79; 95% CI [-1.83, 0.26]; p=0.14; I2 = 0%) (**Figure 3**).

### Secondary outcomes

#### Change from baseline in MADRS score

**Figure 4:**
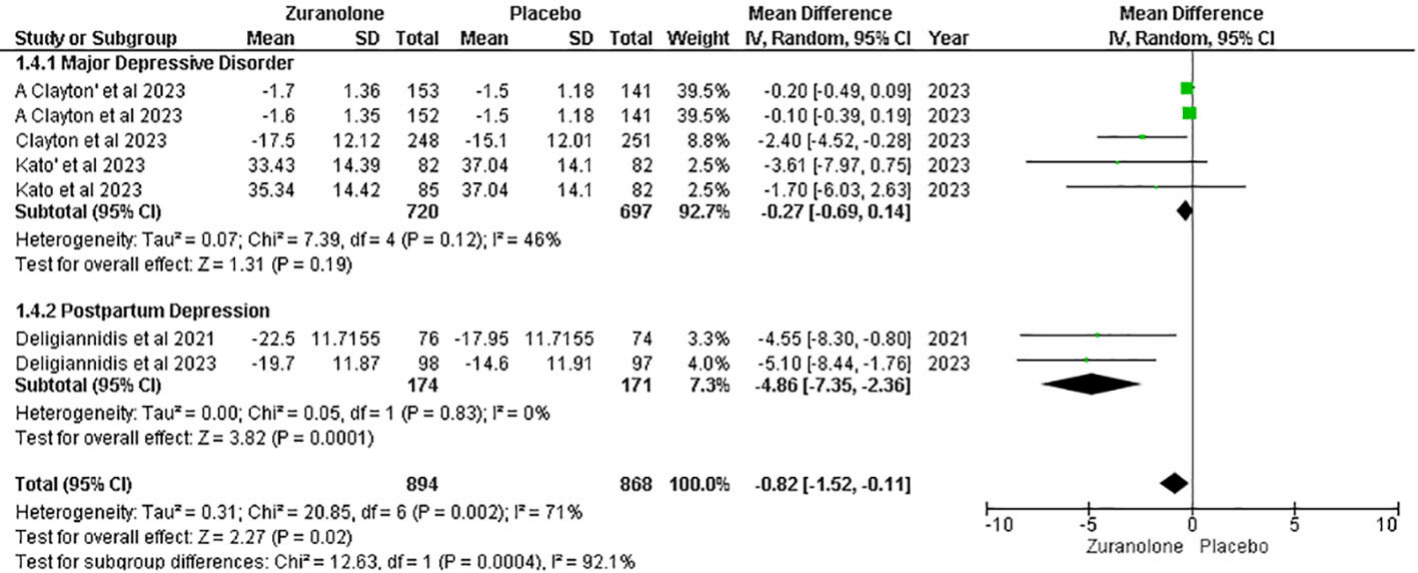
Forest plot of reduction in MADRS score.

Five studies were evaluated to determine the impact of zuranolone on MADRS scores, which demonstrated a decrease in MADRS scores compared to placebo (MD=-0.82; 95% CI= [-1.52, -0.11]; p=0.02; I2 = 71%). A subgroup analysis revealed significant results within the PPD subgroups (MD=-4.86; 95% CI= [-7.35, -2.36]; p=0.0001; I2 = 0). However, no significant findings were observed in the MDD subgroup (MD= -0.27; 95% CI= [-0.69, 0.14]; p=0.12; I2 = 46%) (**Figure 4**).

#### Change from baseline in HAM-A score

**Figure 5:**
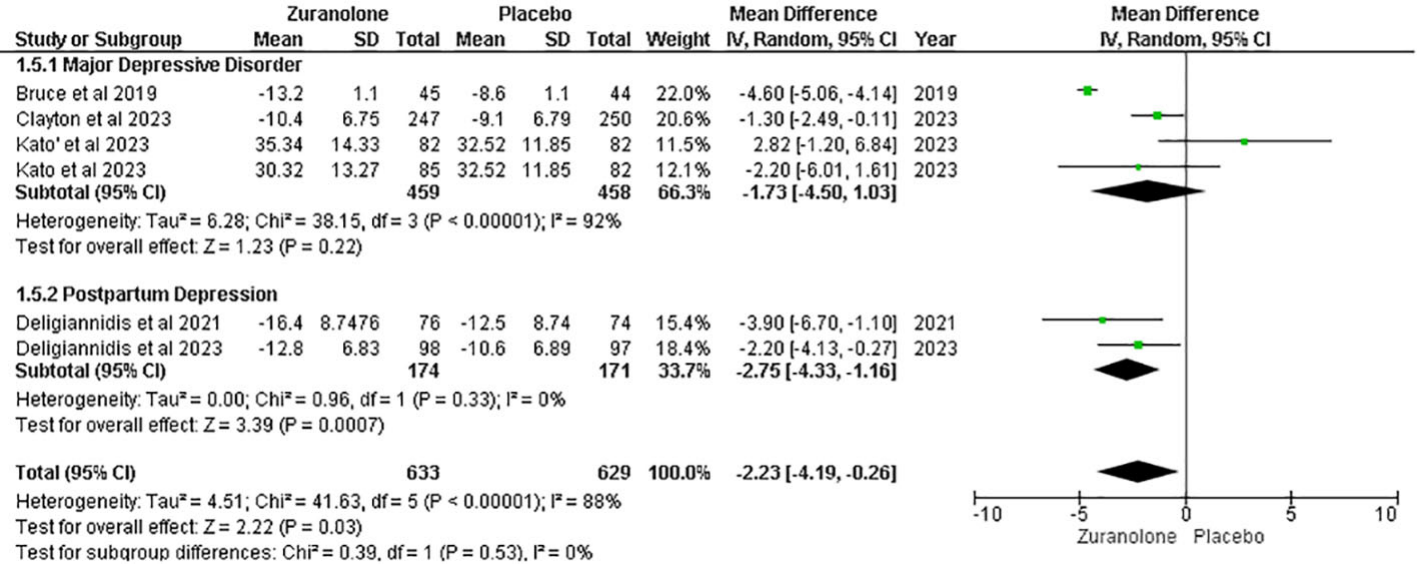
Forest plot of reduction in HAM-A score.

Five studies were evaluated to determine the impact of zuranolone on HAM-A scores, which showed a significant overall reduction in HAM-A scores in patients administered zuranolone compared with placebo (MD=-2.23; 95% CI= [-4.19, -0.26]; p=0.03; I2 = 88%). A subgroup analysis showed significant results in the PPD subgroup (MD -2.75; 95% CI= [-4.33, -1.16]; p=0.0007; I2 = 0%). However, no significant reduction was observed in the MDD subgroup (MD=-1.73; 95% CI= [-4.50, 1.03]; p=0.22; I2 = 92%) (**Figure 5**).

The leave-one-out sensitivity analysis showed that the reduction in the HAM-A score was affected by a single study by Bruce et al. (12). Removing that study resulted in a significant reduction in I2 values (p = 0.09; I2 = 50%) and overall effect [MD = −1.65, 95% CI (−3.17, −0.14), p= 0.03] (**Supplementary Figure 1**).

#### Change from baseline in Bech-6 score

**Figure 6:**
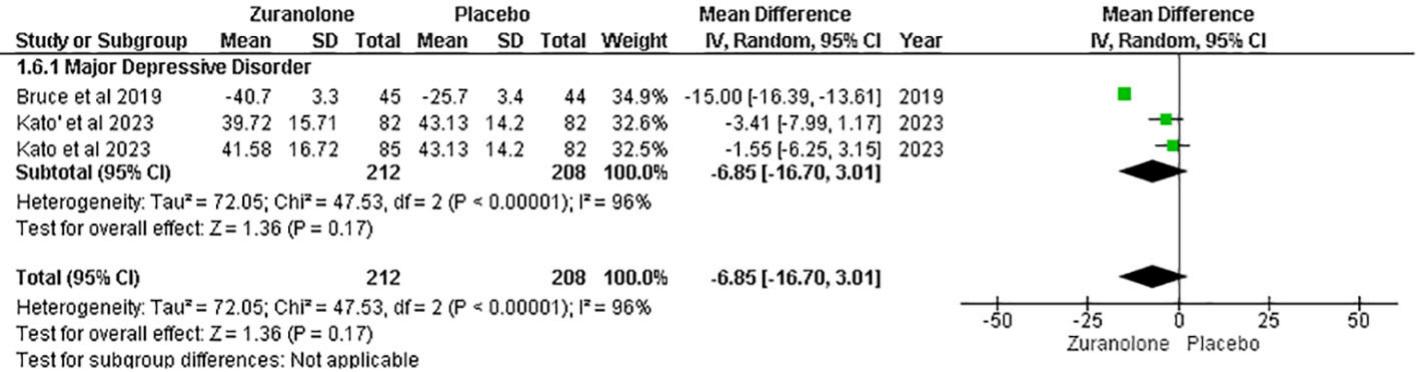
Forest plot of reduction in Bech-6 score.

Two studies were analyzed for the impact of Zuranolone on the Bech-6 score. The results showed a non-significant difference between zuranolone and placebo in reducing the Bech-6 score (MD=-6.85; 95% CI= [-16.70, 3.01]; p=0.17; I2 = 96%). (**Figure 6**) The leave-one-out sensitivity analysis indicated that the Bech-6 score was influenced by a single study, i.e., Bruce et al. (12). When this study was removed, the I2 values showed a significant reduction in overall effect [MD = −2.50, 95% CI (−5.78, 0.78), p = 0.13] (**Supplementary Figure S2**).

#### Treatment-emergent adverse effects

**Figure 7:**
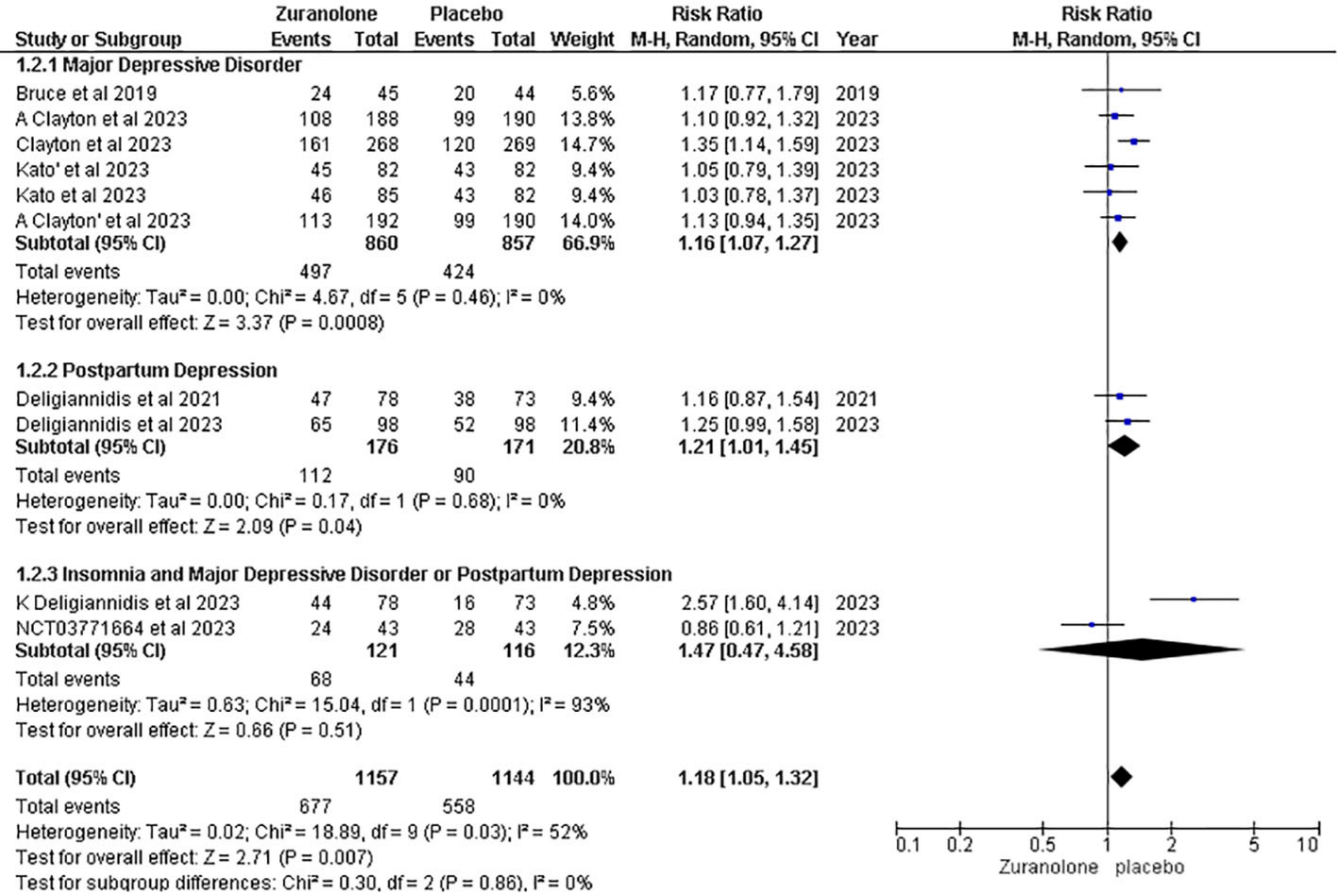
Forest plot of treatment emergent adverse effects.

Eight studies were included for the outcome of TEAEs, and the overall pooled result demonstrated that placebo was associated with fewer TEAEs than Zuranolone. (RR: 1.18, 95% CI [1.05, 1.32], p=0.007; I2 = 52%). After conducting a subgroup analysis, it was found that TEAEs were significantly higher in the Zuranolone group than in the placebo group for MDD and PPD. (RR: 1.16, 95% CI [1.16, 1.27]; p=0.0008, I2 = 0%) and (RR: 1.21, 95% CI [1.01, 1.45]; p=0.04; I2 = 0%) respectively. However, the insomnia subgroup and MDD or PPD did not show a significant association. (RR: 1.47, 95% CI [0.47, 4.58]; p=0.51; I2 = 52%) (**Figure 7**).

#### Serious adverse effects

**Figure 8:**
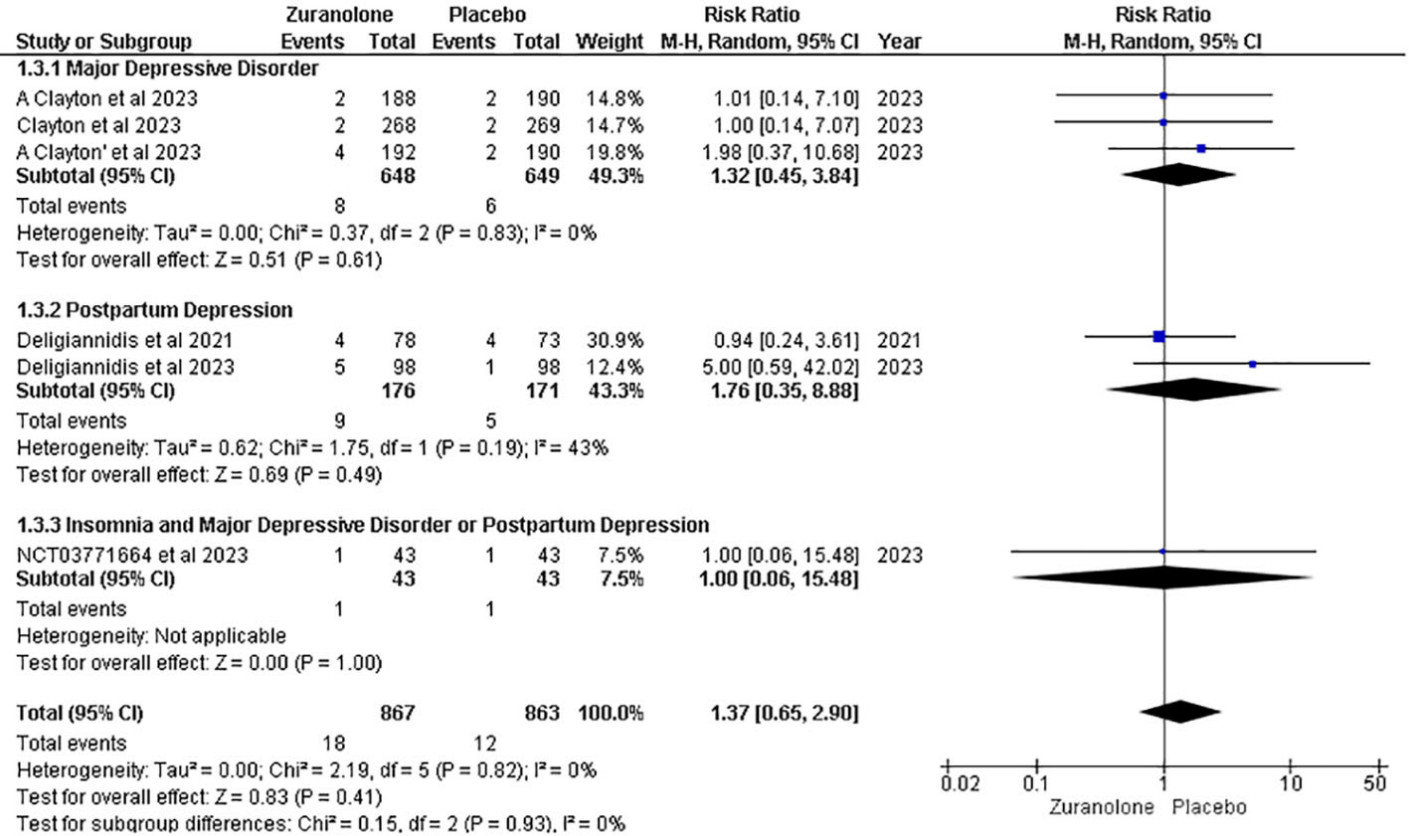
Forest plot of serious adverse effects.

Five studies were included in the analysis of serious adverse effects (AEs), and the overall pooled result showed no significant association between the groups (RR, 1.37; 95% CI [0.65, 2.90], p=0.41; I2 = 0%). Following a subgroup analysis, insignificant findings were revealed for the MDD (RR: 1.32; 95% CI [0.45, 3.84]; p = 0.61; I2 = 0%), PD (RR: 1.76; 95% CI [0.35, 8.88]; p = 0.49; I2 = 43%), and insomnia with MDD or PD (RR: 1.00; 95% CI [0.06, 15.48]; p = 1.00) groups (**Figure 8**).

#### Meta-regression

We assessed mean age, female sex percentage, and body mass index (BMI) as potential covariates that could affect the effect size on our primary outcome and change from baseline in the HAM-D score. Female sex percentage and BMI showed a non-significant association; however, mean age was found to have a significant effect on the HAM-D score. The results are as follows: mean age, Coeff: 0.0270, p=0.0099; female sex percentage, Coeff: -0.0050, p=0.0563; BMI, Coeff: -0.0034, p=0.8616 (**Supplementary Figures 3A–C**).

## Discussion

The purpose of this meta-analysis was to investigate the safety and efficacy of zuranolone in the treatment of postpartum depression, insomnia, and major depressive disorders. Our analysis included eight studies comparing zuranolone with a placebo and focused on six outcomes (24, 25, 30–35). The primary outcome measure was the change in the Hamilton Depression Rating Scale, while the secondary outcomes included the Montgomery Asberg Depression Rating Scale, Hamilton Anxiety Scale, Bech Scale, treatment-emergent side effects, and serious adverse effects. Although our primary outcome and some secondary outcomes showed significant improvements favoring zuranolone, these two outcomes did not reach statistical significance. However, the overall results favored drug use. However, due to the low certainty of the evidence, we approached the findings cautiously, emphasizing the need for additional studies to strengthen the reliability of the results. In summary, our meta-analysis suggests potential benefits of zuranolone in the treatment of postpartum depression, major depressive disorder, and insomnia. Specifically, we observed improvements in HAM-D scores on day 15 with zuranolone compared with placebo, along with reductions in HAM-A and MADRS scores. Nevertheless, zuranolone users experienced mild treatment-related adverse effects.

Depression has been treated with a variety of medications, each presenting a unique balance between efficacy and safety considerations (20). These medications are among the most prescribed for depression, but their efficacy can be limited in certain cases. Tricyclic antidepressants (TCAs) and monoamine oxidase inhibitors (MAOIs) are effective but have notable side effects and carry the risk of toxic reactions if overdosed (36). In contrast, newer drugs such as ketamine have shown promise in treating treatment-resistant depression and reducing suicidal behavior (37). However, concerns regarding its long-term use and potentially severe side effects have tempered enthusiasm. Psilocybin, another emerging option, appears promising for alleviating depression, anxiety, and insomnia (12). However, limited research has hampered broader acceptance (38). These concerns and the quest for more effective and safer treatments culminated in the development of zuranolone, which received FDA approval in 2023 (39). Zuranolone, which acts on GABA receptors, stands out for its rapid onset of action and efficacy in relieving depressive symptoms (6, 40). It helps in the orientation of neuronal circuits and organizes brain function, which is specifically linked to mood and behavior (41).

However, there are concerns regarding its long-term use and safety in the context of MDD; consequently, it has not been approved for use in treating MDD (42, 43). Studies have assessed the efficacy of zuranolone under various conditions. For example, in postpartum depression, significant improvements in the scores of the Hamilton Rating Scale for Depression (HAM-D), the Montgomery-Åsberg Depression Rating Scale (MADRS), and the Hamilton Rating Scale for Anxiety (HAM-A) were observed within a short treatment duration of 14 days, although accompanied by notable adverse effects (34, 44). Additionally, zuranolone has demonstrated benefits in individuals with major depressive disorder by enhancing neural circuits and overall brain function (45). It has also been effective in addressing insomnia in conjunction with postpartum depression, leading to improvements in sleep quality (46, 47). However, the use of zuranolone is associated with side effects, including a higher risk compared with a placebo, as indicated in certain studies (34, 46). Concerns regarding the potential suicide risk and impairment in daily activities have also been raised. Nevertheless, conflicting findings exist, with some studies reporting no significant increase in suicidal ideation, although noting other adverse effects (48). These complexities underscore the ongoing need for comprehensive research and cautious consideration of the benefits and risks of zuranolone in clinical practice.

The levels of zuranolone in the blood were dependent on the dose, with the highest levels observed in the 30 mg dosage group. These dosages were chosen based on safety and tolerability assessments from a prior phase 1 trial involving healthy Japanese individuals, including older adults, where the 30 mg dose showed promising results. Interestingly, both healthy Japanese and White adults had plasma exposure levels that were comparable to those of zuranolone. Furthermore, a phase 2 study conducted in the US on individuals with MDD revealed a significant reduction in depressive symptoms with a 30 mg daily dose of zuranolone over 14 days without encountering any serious safety or tolerability concerns (33, 49). Additionally, a meta-analysis showed that increasing the dose of zuranolone correlated with symptom improvement, although this was accompanied by an increased risk of side effects (34). This finding was supported by another study that demonstrated the dose-dependent efficacy of zuranolone while noting a non-significant association between higher doses and severe adverse effects, consistent with our study’s observations (50). These results were favorable when zuranolone was used for 14 days; however, when the drug was used for a similar duration throughout the year, it led to mild to moderate side effects, which were more common in patients who used 30 mg compared with those who took 50 mg (51).

### Limitations

Despite the insights provided by our study, it has several limitations. First, the limited availability of recent studies resulted in small sample sizes, which could compromise the reliability and accuracy of our findings. Second, insufficient data prevented us from considering preexisting medical conditions that may affect drug efficacy and safety. Lastly, we could not explore the potential interactions between the drug and pretreatment medications, which could influence its efficacy.

## Conclusion

In summary, this meta-analysis was conducted to evaluate the effectiveness of zuranolone in treating MDD and PPD with or without insomnia. The results unequivocally showed a statistically significant improvement in depressive symptoms with the use of zuranolone, which was particularly pronounced on day 15. These findings underscore the potential of zuranolone as a promising therapeutic option for individuals with MDD and PPD with or without insomnia. However, further randomized controlled trials and observational studies are required to gain a deeper understanding of the efficacy of zuranolone in treating MDD and PPD with or without insomnia. It would be highly beneficial if future research included a larger sample size and information about new scales and outcomes.

## Data availability statement

The original contributions presented in the study are included in the article/**Supplementary Material**, further inquiries can be directed to the corresponding author/s.

## Author contributions

AR: Writing – review & editing, Writing – original draft, Visualization, Validation, Supervision, Software, Resources, Project administration, Methodology, Investigation, Formal analysis, Data curation, Conceptualization. SA: Writing – review & editing, Writing – original draft, Visualization, Validation, Software, Resources, Project administration, Methodology, Data curation, Conceptualization. MBAS: Writing – review & editing, Writing – original draft, Visualization, Validation, Supervision, Software, Resources, Project administration, Investigation. SLM: Writing – review & editing, Writing – original draft, Validation, Resources, Project administration, Methodology, Investigation, Conceptualization. RK: Writing – review & editing, Writing – original draft, Visualization, Validation, Supervision, Software, Project administration, Investigation. MA: Writing – review & editing, Writing – original draft, Visualization, Project administration, Methodology, Investigation, Funding acquisition, Data curation, Conceptualization. SR: Writing – review & editing, Writing – original draft, Visualization, Software, Methodology, Investigation, Formal analysis, Data curation. SBA: Writing – review & editing, Writing – original draft, Validation, Methodology, Investigation, Data curation, Conceptualization. HAUR: Writing – review & editing, Writing – original draft, Supervision, Project administration, Methodology, Investigation, Conceptualization. FD: Writing – review & editing, Writing – original draft, Validation, Supervision, Software, Methodology, Investigation, Formal analysis. MA: Writing – review & editing, Writing – original draft, Validation, Supervision, Software, Project administration, Methodology, Investigation.
