# Supplementary material for: Evaluating the safety and efficacy of zuranolone in the management of major depressive disorder and postpartum depression, with or without concurrent insomnia: a rigorous systematic review and meta-analysis
Source: Front Psychiatry. 2024 Jul 5;15:1425295. doi: 10.3389/fpsyt.2024.1425295 (PMC11257908; doi:10.3389/fpsyt.2024.1425295)
Supplement: Supplementary file 1 [file DataSheet_1.docx]

**Supplementary Table 1.** Detailed search strategy used in each database.

| **Search Strategy** | **Database** | **Citations** |  |
| --- | --- | --- | --- |
|  |  |  |  |
| (((zuranolone OR sage-217)) AND ((post partum depression))) AND ((major depressive disorders)) | **PUBMED** | 16 |  |
|  |  |  |  |
| (zuranolone* OR sage-217*) AND (postpartum depression* OR major depressive disorders*) | **SCOPUS** | 147 |  |
|  |  |  |  |
| Condition or disease; Postpartum Depression OR Major Depressive Disorders Intervention or treatment; zuranolone OR SAGE-217 | **CLINICAL TRIALS.GOVT** | 11 |  |
|  |  |  |  |
| #1 zuranalone  #2 major depressive disorder #3 postpartum depression #1 AND #2 AND #3 | **COCHRANE LIBRARY** | 5 |  |
|  |  |  |  |
| (zuranolone OR sage-217) AND (postpartum depression OR major depressive disorders) | **SPRINGER LINKS** | 60 |  |
|  |  |  |  |
| zuranolone OR sage-217 AND post partum depression AND major depressive disorders | **EUROPE PMC** | 86 |  |
|  |  |  |  |
| zuranolone AND post partum depression OR major depressive disorders | **GOOGLE SCHOLAR** | 299 |  |
|  |  |  |  |

**Supplementary Table 2**. Risk of Bias Assessment Table with Reasons

|  | Cochrane Risk-of-Bias Tool | | |
| --- | --- | --- | --- |
|  | Bias | Risk of bias | Author judgement |
| A Clayton et al 2023 | Random sequence generation (selection bias) | Low Risk | This study (MOUNTAIN; NCT03672175) was a randomized, double-blind, parallel-group, placebocontrolled, phase 3 trial in patients with MDD conducted at 55 sites across the United States |
|  | Allocation concealment (selection bias) | Low Risk | Zuranolone 30 mg dose was selected based on a phase 2 study in patients with MDD.54 The lower, once-daily 20 mg dose was included to assess for minimal effective dose. Dose adjustments were not permitted. |
|  | Blinding of participants and personnel (performance bias) | Low Risk | The participants were blinded. |
|  | Blinding of outcome assessment (detection bias) | Unclear Risk | Insufficient detail to allow definitive judgement. |
|  | Incomplete outcome data (attrition bias) | Low Risk | No missing outcome data. |
|  | Selective reporting (reporting bias) | Low Risk | All pre-specified endpoints were reported. |
|  | Other bias | Low Risk | The study appears to be free of other sources of bias. |
| Bruce et al 2019 | Random sequence generation (selection bias) | Low Risk | Randomization was performed with the use of interactiveresponse technology created by 4G Clinical (Wellesley, MA). |
|  | Allocation concealment (selection bias) | Unclear Risk | Insufficient detail to allow definitive judgement.” |
|  | Blinding of participants and personnel (performance bias) | Unclear Risk | Insufficient detail to allow definitive judgement.” |
|  | Blinding of outcome assessment (detection bias) | Unclear Risk | Insufficient detail to allow definitive judgement. |
|  | Incomplete outcome data (attrition bias) | Low Risk | No missing outcome data. |
|  | Selective reporting (reporting bias) | Low Risk | All pre-specified endpoints were reported. |
|  | Other bias | Low Risk | The study appears to be free of other sources of bias. |
| Clayton et al 2023 | Random sequence generation (selection bias) | Low Risk | Patients were randomized to one of the two groups, using computer-generated random-number assignment. |
|  | Allocation concealment (selection bias) | Unclear Risk | Insufficient detail to allow definitive judgement. |
|  | Blinding of participants and personnel (performance bias) | Low Risk | Patients were blinded |
|  | Blinding of outcome assessment (detection bias) | Unclear Risk | Insufficient detail to allow definitive judgement. |
|  | Incomplete outcome data (attrition bias) | Low Risk | No missing outcome data. |
|  | Selective reporting (reporting bias) | Low Risk | All pre-specified endpoints were reported. |
|  | Other bias | Low Risk | The study appears to be free of other sources of bias. |
| Deligiannidis et al 2021 | Random sequence generation (selection bias) | Low Risk | Randomization codes were generated with a block size of 4 by an independent statistical vendor not affiliated with Sage Therapeutics, Inc.. |
|  | Allocation concealment (selection bias) | Unclear Risk | Insufficient detail to allow definitive judgement. |
|  | Blinding of participants and personnel (performance bias) | Low Risk | This phase 3, double-blind, randomized, outpatient, placebo-controlled clinical trial was conducted between January 2017 and December 2018 in 27 enrolling US sites. |
|  | Blinding of outcome assessment (detection bias) | Unclear Risk | Insufficient detail to allow definitive judgement. |
|  | Incomplete outcome data (attrition bias) | Low Risk | Quote: “No missing outcome data.” |
|  | Selective reporting (reporting bias) | Low Risk | All pre-specified endpoints were reported. |
|  | Other bias | Low Risk | The study appears to be free of other sources of bias. |
| Deligiannidis et al 2023 | Random sequence generation (selection bias) | Low Risk | Patients were randomized in a 1:1 ratio and in a stratified manner based on antidepressant use (current, stable use vs. not treated or withdrawn from antidepressants for $30 days or .5 half-lives) to receive zuranolone 50 mg/day or placebo. Patients, |
|  | Allocation concealment (selection bias) | Low Risk | Patients self-administered zuranolone 50 mg/day or placebo orally once daily in the evening with fat-containing food for 14 days. Study drug administration was monitored by a smartphone medical adherence monitoring platform to visually confirm ingestion |
|  | Blinding of participants and personnel (performance bias) | Low Risk | clinicians, and study personnel were blinded to treatment allocation during the study |
|  | Blinding of outcome assessment (detection bias) | Unclear Risk | Insufficient detail to allow definitive judgement. |
|  | Incomplete outcome data (attrition bias) | Low Risk | No missing outcome data. |
|  | Selective reporting (reporting bias) | Low Risk | All pre-specified endpoints were reported. |
|  | Other bias | Low Risk | The study appears to be free of other sources of bias. |
| Kato et al 2023 | Random sequence generation (selection bias) | Low Risk | patients were randomized (1:1:1) at baseline (visit 1), with stratification based on the 17-item Hamilton Depression Rating Scale (HAMD-17) total score at baseline. |
|  | Allocation concealment (selection bias) | Unclear Risk | Insufficient detail to allow definitive judgement. |
|  | Blinding of participants and personnel (performance bias) | Low Risk | double-blind, placebo-controlled, parallel-group study was conducted in patients with MDD at 72 sites in Japan |
|  | Blinding of outcome assessment (detection bias) | Unclear Risk | Insufficient detail to allow definitive judgement. |
|  | Incomplete outcome data (attrition bias) | Low Risk | No missing outcome data. |
|  | Selective reporting (reporting bias) | Low Risk | All pre-specified endpoints were reported. |
|  | Other bias | Low Risk | The study appears to be free of other sources of bias. |
| K Deligiannidis et al 2023 | Random sequence generation (selection bias) | Low Risk | This phase 3, double-blind, randomized, outpatient, placebo-controlled clinical trial was conducted between January 2017 and December 2018 in 27 enrolling US sites. |
|  | Allocation concealment (selection bias) | Unclear Risk | Insufficient detail to allow definitive judgement. |
|  | Blinding of participants and personnel (performance bias) | Unclear Risk | This phase 3, double-blind, randomized, outpatient, placebo-controlled clinical trial was conducted between January 2017 and December 2018 in 27 enrolling US sites. |
|  | Blinding of outcome assessment (detection bias) | Unclear Risk | Insufficient detail to allow definitive judgement. |
|  | Incomplete outcome data (attrition bias) | Low Risk | No missing outcome data. |
|  | Selective reporting (reporting bias) | Low Risk | All pre-specified endpoints were reported. |
|  | Other bias | Low Risk | The study appears to be free of other sources of bias. |
| NCT03771664 et al 2023 | Random sequence generation (selection bias) | Low Risk | Patients was randomized. |
|  | Allocation concealment (selection bias) | High Risk | Participants received SAGE-217 matching placebo capsules (single-blind), orally, once daily prior to Day 1 (Days -2 and -1) followed by self-administration of SAGE-217, 30 milligrams (mg) capsules, orally, once daily for 12 days. Thereafter participants received SAGE-217, 30 mg capsules, orally, once daily, 30 minutes prior to lights out (PSG) on Days 13 and 14 (double-blind). Thereafter participants self-administered SAGE-217 matching placebo capsules (single-blind), orally, once daily on Days 15 to 21. |
|  | Blinding of participants and personnel (performance bias) | Low Risk | Randomized, Double-blind, Placebo-controlled Study of the Safety, |
|  | Blinding of outcome assessment (detection bias) | Unclear Risk | Insufficient detail to allow definitive judgement. |
|  | Incomplete outcome data (attrition bias) | Low Risk | No missing outcome data. |
|  | Selective reporting (reporting bias) | Low Risk | All pre-specified endpoints were reported. |
|  | Other bias | Low Risk | The study appears to be free of other sources of bias. |

**Supplementary Figure 1. Leave One Out Analysis Forest Plot of Change from baseline in HAM-A score**


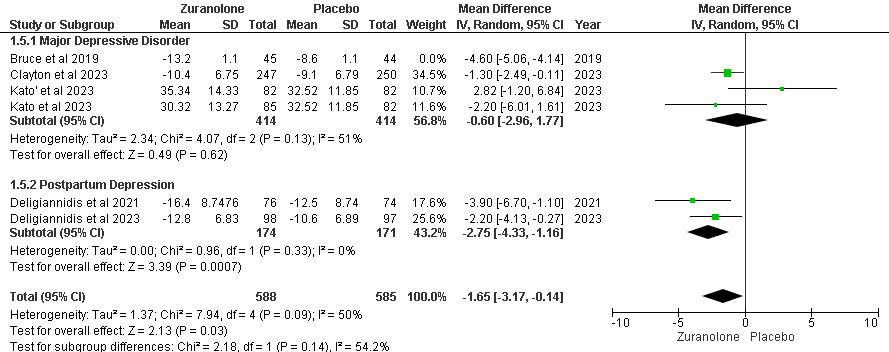


**Supplementary Figure 2. Leave One Out Analysis Forest Plot of Change from baseline in Bech-6 score**

**
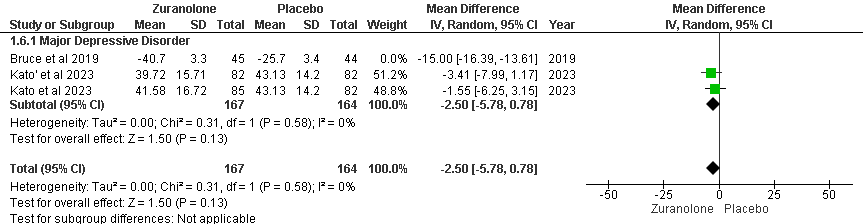
**

**Supplementary Figure 3A.** Scatter plot for mean age

**Supplementary Figure 3B.** Scatter plot for female sex %


**Supplementary Figure 3C.** Scatter plot for BMI
